# Across the Baltic: a new record for an enigmatic black scavenger fly, *Zuskamira inexpectata* (Pont, 1987) (Sepsidae) in Finland

**DOI:** 10.3897/BDJ.3.e4308

**Published:** 2015-06-26

**Authors:** Yuchen Ang, Patrick Thomas Rohner, Rudolf Meier

**Affiliations:** ‡ National University of Singapore, Singapore, Singapore; § University of Zurich, Zurich, Switzerland

**Keywords:** Diptera, Sepsidae, Zuskamira, Finland, Sweden, Germany, Fennoscandia, new record

## Abstract

Specimens of the enigmatic, monotypic European genus *Zuskamira* Pont, 1987 (Sepsidae) were initially collected only from the lower central Swedish provinces of Darlana, Uppland and Västmanland. However, the same species was subsequently found much more south in Lower-Saxony and Schleswig-Holstein although Germany is overall well sampled for sepsid flies. Here we report a further (longitudinal) range expansion based on new localities in Southern Finland. New localities for Finland and Sweden are here added and we discuss briefly the habitat requirements of the species.

## Introduction

Sepsidae (Acalyptratae: Cyclorrhapha), or ant-like scavenger flies, form a small to medium-sized, cosmopolitan, family-ranked clade of saprophagous flies with ca. 370 described species (Ozerov 2005). One of the more surprising finds of the last 30 years was the discovery of two new monotypic genera in Europe (*Zuskamira*, *Susanomira*) of which *Zuskamira* was until recently only known from a few localities in Sweden (Pont 1987). More recently, the species was also found in Germany (Stuke 2005) which was surprising because this country is overall fairly well sampled for Sepsidae (Pont and Meier 2002). *Zuskamira* was described by Pont (1987) and currently contains one species only, viz. *Z. inexpectata* Pont, 1987. The species is morphologically distinct from other sepsids, based on the following male characters: (1) 4^th^ sternite heavily spinose, (2) syn-sternite 7+8 absent, (3) epandrium enlarged, and (4) hypandrium firmly attached to syntergite 7+8 at one point (Pont 1987). Subsequent phylogenetic research (Zhao et al. 2013, Su et al. 2008, Laamanen et al. 2005, Pont and Meier 2002) showed that this morphological distinctness is associated with a similarly distinct phylogenetic position on the phylogenetic tree of Sepsidae: *Zuskamira inexpectata* is not nested within any other sepsid genus, and behaves like a "wildcard" taxon whose actual placement on the sepsid phylogeny remains unclear because different topologies are supported by the same data depending on how the data are analyzed (Zhao et al. 2013).

*Zuskamira inexpectata* is overall a rare and elusive species, and has previously only been recorded in small numbers on horse dung in the lower central parts of Sweden (Darlana, Uppland and Västmanland) and more recently in Northern Germany (Lower Saxony and Schleswig-Holstein). Herein, we report its presence in Southern Finland and add several additional Swedish localities to the known distribution. Overall, this suggests that the species distribution is larger than previously recognized or the species is undergoing a range expansion.

## Materials and methods

Specimens were collected during two field collection trips in 2011 and 2014. *Zuskamira inexpectata* is very closely associated with horse dung, so horse farms and riding centers were targeted. Specimens were caught via sweep-netting; by slowly approaching a pile of dung and quickly placing the net over the dung pat, inducing the flies to walk upwards towards the end of the net (Fig. 2​). Sweeping surrounding vegetation yielded very few specimens; it is likely that the species tend to hide very low in the vegetation.

Of the caught specimens, some were immediately stored in 70% alcohol and others ground up in RNA-later for further transcriptomic work. Specimens were identified based on the key given in Pont and Meier (2002) and two alcohol specimens (1 ♂ 1 ♀) from the Finnish locality Uusimaa (see Other Materials tab C in the Taxon Treatment section) were imaged with the Visionary Digital Lab+ photomicrography system (at "CF4-P3" magnification). Their habitus images are provided in the results section. These specimens are deposited in the Lee Kong Chian Natural History Museum (formerly Raffles Museum of Biodiversity Research, in Singapore). Genbank records for the species can be found here: http://www.ncbi.nlm.nih.gov/Taxonomy/Browser/wwwtax.cgi?id=192462

## Taxon treatments

### Zuskamira inexpectata

Pont, 1987

http://sepsidnet-rmbr.nus.edu.sg/Zuskamira_inexpectata.html

#### Materials

- **Type status:** Holotype. **Occurrence:** recordedBy: Adrian Pont; individualCount: 1; sex: Males and Females; lifeStage: adult; **Taxon:** scientificName: Zuskamira inexpectata; family: Sepsidae; genus: Zuskamira; specificEpithet: inexpectata; taxonRank: species; scientificNameAuthorship: Pont, 1987; **Location:** country: Sweden; countryCode: SE; stateProvince: Västmanland; county: Örebro; municipality: Nora Municipality; locality: Klacka Leberg; verbatimLocality: Västmanland, Klacka Leberg; georeferenceSources: label; **Identification:** identificationID: Zuskamira inexpectata; identifiedBy: Adrian Pont; dateIdentified: 1987; **Event:** samplingProtocol: Sweep-netting; eventDate: 22.vi.1986; habitat: Horse-pasture; **Record Level:** language: en; institutionID: Natural History Museum (formerly British Museum of Natural History); institutionCode: NHML (formerly BMNH); collectionCode: BMNH(E) 1239011
- **Type status:** Paratype. **Occurrence:** recordedBy: Adrian Pont; individualCount: 2; sex: Male; lifeStage: adult; **Taxon:** scientificName: Zuskamira inexpectata; family: Sepsidae; genus: Zuskamira; specificEpithet: inexpectata; taxonRank: species; scientificNameAuthorship: Pont, 1987; **Location:** country: Sweden; countryCode: SE; stateProvince: Dalarna; county: Dalarna; municipality: Malung-Sälen Municipality; locality: Sälen; verbatimLocality: Dalarna, Sälen District, 3km west of Horrmund; georeferenceSources: label; **Identification:** identificationID: Zuskamira inexpectata; identifiedBy: Adrian Pont; dateIdentified: 1987; **Event:** samplingProtocol: Sweep-netting; eventDate: 5.vii.1986; habitat: Horse-pasture; **Record Level:** language: en; institutionID: Zoological Museum University Copenhagen; institutionCode: ZMUC
- **Type status:** Paratype. **Occurrence:** recordedBy: Adrian Pont; individualCount: 1; sex: Male; lifeStage: adult; **Taxon:** scientificName: Zuskamira inexpectata; family: Sepsidae; genus: Zuskamira; specificEpithet: inexpectata; taxonRank: species; scientificNameAuthorship: Pont, 1987; **Location:** country: Sweden; countryCode: SE; stateProvince: Uppland; county: Uppsala; municipality: Tierp; locality: Tierp; verbatimLocality: Uppland, near Tierp; georeferenceSources: label; **Identification:** identificationID: Zuskamira inexpectata; identifiedBy: Adrian Pont; dateIdentified: 1987; **Event:** samplingProtocol: Sweep-netting; eventDate: 9.vii.1962; habitat: Horse-pasture; **Record Level:** language: en; institutionID: Natural History Museum, London (formerly British Museum of Natural History); institutionCode: NHML (formerly BMNH); collectionCode: BMNH
- **Type status:** Other material. **Occurrence:** recordedBy: Rudolf Meier; individualCount: 1; sex: Male; lifeStage: adult; **Taxon:** scientificName: Zuskamira inexpectata; family: Sepsidae; genus: Zuskamira; specificEpithet: inexpectata; taxonRank: species; scientificNameAuthorship: Pont, 1987; **Location:** country: Sweden; countryCode: SE; stateProvince: Dalarna; county: Dalarna; municipality: Malung-Sälen Municipality; locality: Lima; verbatimLocality: Dalarna, Lima; decimalLatitude: 60.93741; decimalLongitude: 13.36398; georeferenceSources: GPS; **Identification:** identificationID: Zuskamira inexpectata; identifiedBy: Rudolf Meier; dateIdentified: 1991; **Event:** samplingProtocol: Sweep-netting; eventDate: vi.1991; habitat: Horse-pasture; **Record Level:** language: en; institutionID: Lee Kong Chian Natural History Museum (LKCNHM, formerly Raffles Museum of Biodiversity Research); institutionCode: LKCNHM (formerly RMBR)
- **Type status:** Other material. **Occurrence:** recordedBy: Yuchen Ang, Rudolf Meier, Patrick Rohner; individualCount: 20; sex: Males(12) and Females(8); lifeStage: adult; **Taxon:** scientificName: Zuskamira inexpectata; family: Sepsidae; genus: Zuskamira; specificEpithet: inexpectata; taxonRank: species; scientificNameAuthorship: Pont, 1987; **Location:** country: Sweden; countryCode: SE; stateProvince: Dalarna; county: Dalarna; municipality: Orsa; locality: Orsa; verbatimLocality: Dalarna, Orsa; decimalLatitude: 61.12278; decimalLongitude: 14.48222; georeferenceSources: GPS; **Identification:** identificationID: Zuskamira inexpectata; identifiedBy: Yuchen Ang; dateIdentified: 2014; **Event:** samplingProtocol: Sweep-netting; eventDate: 7.vii.2014; habitat: Horse-pasture; **Record Level:** language: en; institutionID: Lee Kong Chian Natural History Museum (LKCNHM, formerly Raffles Museum of Biodiversity Research); institutionCode: LKCNHM (formerly RMBR)
- **Type status:** Other material. **Occurrence:** recordedBy: Yuchen Ang; individualCount: 2; sex: Male and Female; lifeStage: adult; **Taxon:** scientificName: Zuskamira inexpectata; family: Sepsidae; genus: Zuskamira; specificEpithet: inexpectata; taxonRank: species; scientificNameAuthorship: Pont, 1987; **Location:** country: Finand; countryCode: FI; stateProvince: Uusimaa; county: Uusimaa; municipality: Lohja; locality: Lohja; verbatimLocality: Southern Finland, Uusimaa, sub-Helsinki, Lohja; decimalLatitude: 60.26333; decimalLongitude: 24.23444; georeferenceSources: GPS; **Identification:** identificationID: Zuskamira inexpectata; identifiedBy: Yuchen Ang; dateIdentified: 2011; **Event:** samplingProtocol: Sweep-netting; eventDate: 20.viii.2011; habitat: Horse-pasture; **Record Level:** language: en; institutionID: Lee Kong Chian Natural History Museum (LKCNHM, formerly Raffles Museum of Biodiversity Research); institutionCode: LKCNHM (formerly RMBR)

#### Distribution

Sweden, Finland, Germany.

#### Ecology

Saprophagous species, obligate breeder on horse dung.

#### Taxon discussion

An excellent description of *Z. inexpectata* was provided by Pont (1987) and further discussed in relation to other sepsid genera in Pont and Meier 2002. The specimens from Finland and Sweden fit the descriptions well, but we are here nevertheless providing a high-resolution image of the male lateral habitus (Fig. 3a) and ventral abdomen (Fig. 3b) based on specimens acquired from Finland in 2014 because such images can serendipitously capture morphological differences that may become important in future research Ang et al. 2013a. This image is also placed on the digital reference collection for the Sepsidae, Sepsidnet Ang et al. 2013b​.

*Zuskamira inexpectata* appears to have fairly specific habitat requirements while many other sepsid species are generalists, are found in many habitats, and can be bred on bovine dung (Pont and Meier 2002). Exceptions include *Orygma luctuosum* Meigen, 1830 which only breeds on beached kelp-wrack and some *Themira* species that only breed on waterfowl dung (Pont and Meier 2002). *Zuskamira inexpectata* appears to be an obligate specialist for horse dung. This is shared with *Ortalischema albitarse* Zetterstedt, 1847 while *Susanomira caucasica* is now also known from cow dung (pers. comm., A. Pont). Another unusual life history feature of *Z. inexpectata* is that the puparia require a winter for diapause before the adult stage emerges (Pont and Meier 2002). The narrow choice of substrate and habitat may explain why *Z. inexpectata* appears overall fairly rare and why only two specimens were caught in Finland in late August 2011. This is also reflected in the low numbers recorded by Stuke (2005) in Germany.

**Figure 1.**
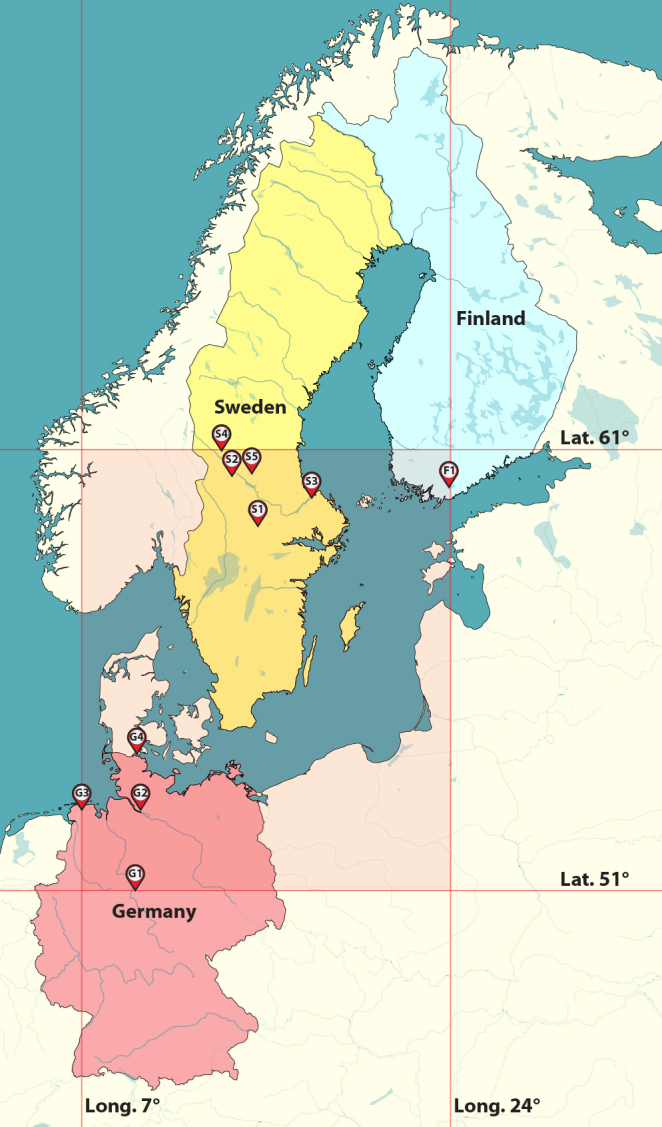
Map of Fennoscandia showing collection localities for *Zuskamira inexpectata* in Sweden: (S1) Klacka-Lerberg, (S2) Sälen, (S3) Tierp, (S4) Lima and (S5) Orsa; in Germany: (G1) Nieste, (G2) "Holmer Fischteiche", (G3) Logaerfeld and (G4) Flensburg; and in Finland: (F1) Lohja. Detailed locality information in results section. Semi-transparent red lines represent the borders of the latitudinal and longitudinal bands in which *Z. inexpectata* has been found.

Initially, *Z. inexpectata* had a very limited known distribution, lying within Sweden in a narrow 59°N - 61°N latitudinal band from Klacka-Lerberg to Sälen (Pont 1987). The findings by Stuke (2005) expanded the species limits ca. 900km south to 51°N (Nieste, Germany). Our findings further expand *Z. inexpectata*’s distribution latitudinally, across the Baltic Sea into Southern Finland (Fig. 1).

It is possible that *Z. inexpectata* is actually currently expanding its distribution, given that European Sepsidae has been extensively sampled (Pont and Meier 2002), and no *Z. inexpectata* have been found outside of Sweden until 2005. Climate has often been an effective barrier for dispersal (e.g., McGlynn 1999, Ang et al. 2008, Kobelt and Nentwig 2008, Smith et al. 2007), but it is not the case here: the Finnish locality is within the latitudinal range of the Swedish distribution and has a similar climate, while the German localities do not have drastically different (and in fact, milder) climates than the localities in Sweden and Finland.

As mentioned earlier, *Z. inexpectata* is dependent on horse dung for breeding - one may speculate that the species is being spread to new areas as a synanthrophic commensal alongside equestrian activities, given that humans commonly transport arthropods to new areas (McGlynn 1999, Smith et al. 2007, Ang et al. 2008, Kobelt and Nentwig 2008). It is thus conceivable that *Z. inexpectata* may eventually be found in other countries in Western Europe and Scandinavia.​

#### Notes

Based on his original description, Pont (1987) collected *Z. inexpectata* from (S1) Klacka-Lerberg (Västmanland, 22.vi.1986), (S2) in Sälen district, “3km west of Horrmund” (Dalarna, 5.vii.1986) and (S3) near Tierp (Uppland, 9.vii.1962). RM collected additional specimens from (S4) Lima (Dalarna, vi.1999). Surprisingly, Stuke (2005) also recorded additional specimens in the German states of Lower Saxony, from (G1) "Endschlagbach 1 km W Nieste", (G2) Holmer Fischteiche and (G3) Leer, Logaerfeld as well as (G4) "E Flensburg Rothenhaus, NF3375" in Schleswig-Holstein. In 2011, a field collection trip by Ang in Southern Finland yielded two specimens of *Z. inexpectata* from (F1) a horse farm in Lohja (Uusimaa, sub-Helsinki region. A subsequent field collection trip by Ang to the same locality on 28.vii.2014 yielded many additional specimens. The same trip also yielded *Z. inexpectata* from (S5) Orsa County (Dalarna) in Sweden. These records currently constitute all the known collecting localities for *Z. inexpectata* (Fig. 1).

**Figure 2.**
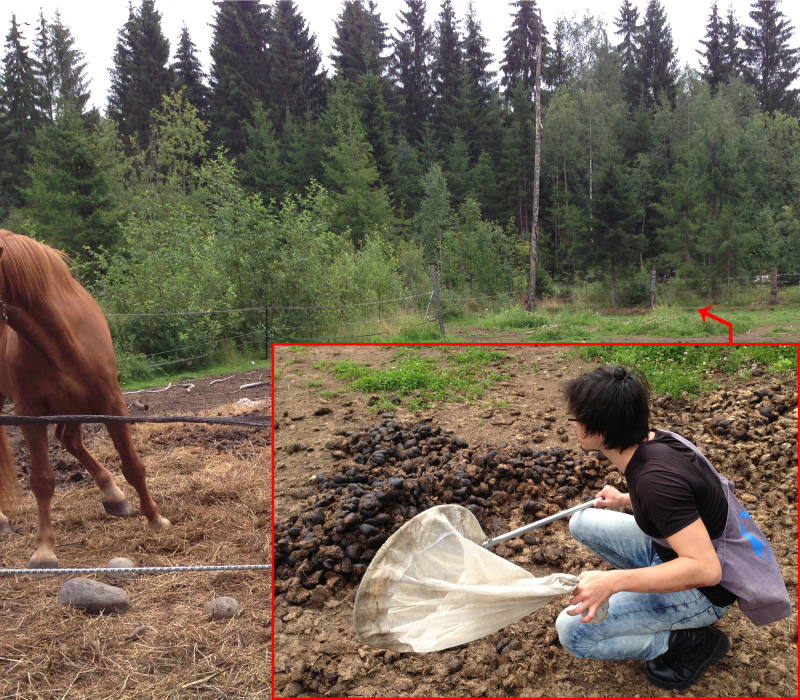
Photo showing habitat of collection locality in Southern Finland. Red inset shows the particular dungpile where numerous *Zuskamira inexpectata* were collected; note that it is at the edge of the pasture and close-by to shrubbery.

Despite exploring numerous (including previously sampled) pastures, only one pasture in Sweden yielded small numbers of *Z. inexpectata* specimens during our 2014 collecting trip. The collected specimens were almost always seen on the horse dung itself or in the immediate surrounding vegetation. Sweep-netting of vegetation even a meter away from horse dung almost never yielded this species. The microhabitat was also specific: only fairly fresh piles of horse dung (i.e., still moist on the exterior) in moist -but not flooded- pastures with relatively short grassy vegetation or hay, and close-by to wooded or bushy areas (see Fig. 2) yielded specimens. Furthermore, specimens were collected only during sunny, warmer periods of the day. Overall, the species initially appeared fairly rare in Finland when only two specimens were collected while other sepsids, such as *Themira annulipes* (Meigen 1826), were very common. However, during the trip in July 2014 the reverse was found as *Z. inexpectata* was fairly common while *T. annulipes* was rare.​

An updated checklist based on previous records (Pont and Meier 2002, Ozerov 2005, Kahanpää and Winqvist 2014) indicates that Finland is home to 33 species of Sepsidae across 7 genera (see Table 1​ for list of species).

## Supplementary Material

XML Treatment for Zuskamira inexpectata

## Figures and Tables

**Figure 3a.**
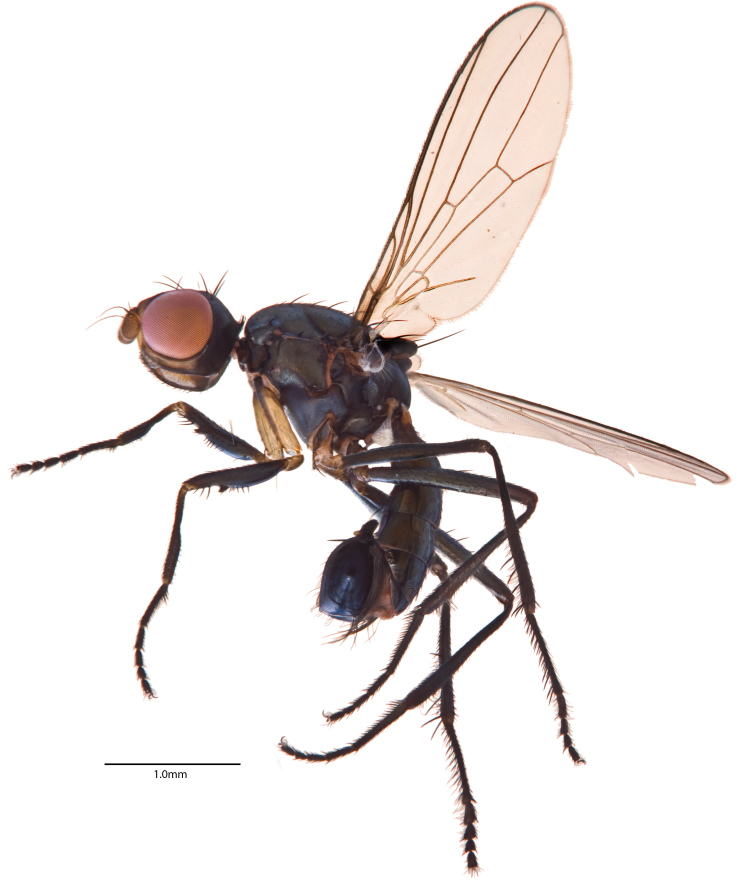
Lateral habitus

**Figure 3b.**
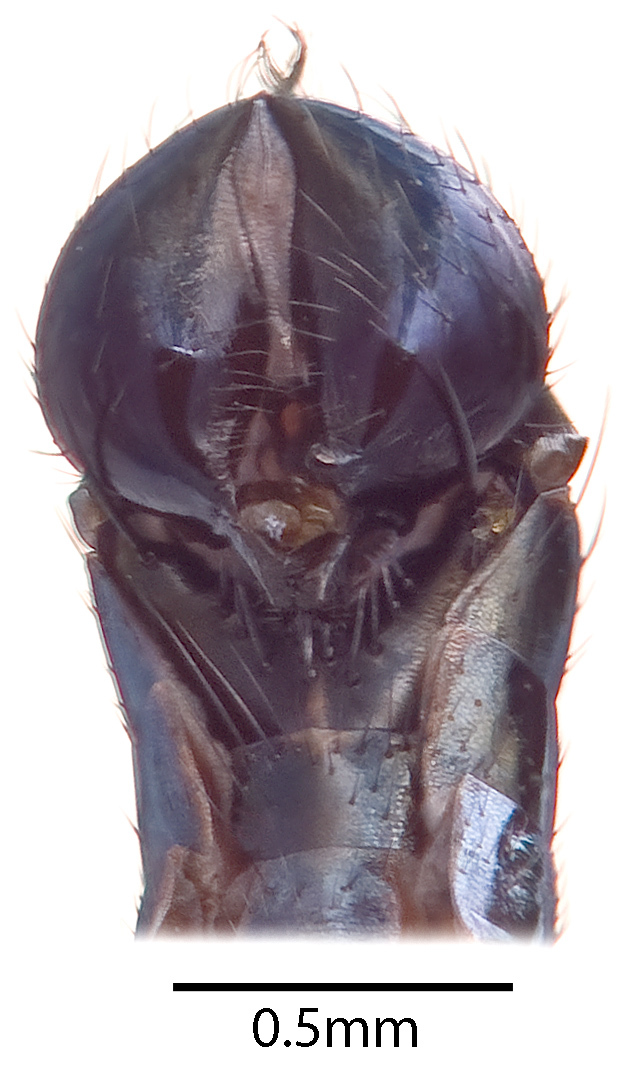
Ventral abdomen (posterior section)

**Table 1.** List of species of Sepsidae known across Finland.

***Meroplius* Rondani, 1874 (2 spp.)**
*Meroplius minutus* (Wiedemann, 1830)
*Meroplius fukuharai* (Iwasa, 1984)
***Nemopoda* Robineau-Desvoidy, 1830 (3 spp.)**
*Nemopoda nitidula* (Fallén, 1820)
*Nemopoda pectinulata* Loew, 1873
*Nemopoda speiseri* (Duda, 1926)
***Saltella* Robineau-Desvoidy, 1830 (1 sp.)**
*Saltella sphondylii* (Schrank, 1803)
***Sepsis* Fallén, 1810 (11 spp.)**
*Sepsis biflexuosa* Strobl, 1893
*Sepsis cynipsea* (Linnaeus, 1758)
*Sepsis duplicata* Haliday, 1838
*Sepsis flavimana* Meigen, 1826
*Sepsis fulgens* Meigen, 1826
*Sepsis luteipes* Melander et Spuler, 1917
*Sepsis nigripes* Meigen, 1826
*Sepsis orthocnemis* Frey, 1908
*Sepsis punctum* (Fabricius, 1794)
*Sepsis thoracica* (Robineau-Desvoidy, 1830)
*Sepsis violacea* Meigen, 1826
***Themira* Robineau-Desvoidy, 1830 (14 spp.)**
*Themira annulipes* (Meigen, 1826)
*Themira arctica* (Becker, 1915)
*Themira biloba* Andersson, 1975
*Themira germanica* Duda, 1926
*Themira gracilis* (Zetterstedt, 1847)
*Themira leachi* (Meigen, 1826)
*Themira lucida* (Staeger, 1844)
*Themira malformans* Melander et Spuler, 1917
*Themira minor* (Haliday, 1833)
*Themira nigricornis* (Meigen, 1826)
*Themira paludosa* Elberg, 1963
*Themira pusilla* (Zetterstedt, 1847)
*Themira putris* (Linnaeus, 1758)
*Themira superba* (Haliday, 1833)
***Ortalischema* Frey 1925 (1 sp.)**
*Ortalischema albitarse* (Zetterstedt, 1847)
***Zuskamira* Pont 1987 (1 sp.)**
*Zuskamira inexpectata* Pont, 1987
**Total: 33 spp.**
